# “We Will Always Ask Ourselves the Question of How to Feed the Family”: Subsistence Farmers’ Perceptions on Adaptation to Climate Change in Burkina Faso

**DOI:** 10.3390/ijerph17197200

**Published:** 2020-10-01

**Authors:** Raissa Sorgho, Isabel Mank, Moubassira Kagoné, Aurélia Souares, Ina Danquah, Rainer Sauerborn

**Affiliations:** 1 Heidelberg Institute of Global Health (HIGH), Heidelberg University Hospital, Heidelberg University, Im Neuenheimer Feld 324, 69120 Heidelberg, Germany; isabel.mank@uni-heidelberg.de (I.M.); aurelia.souares@uni-heidelberg.de (A.S.); ina.danquah@uni-heidelberg.de (I.D.); 2 Centre de Recherche en Santé de Nouna (CRSN), Ministry of Health, Nouna P.O. Box 02, Burkina Faso; kmoubache@yahoo.fr

**Keywords:** climate change, adaptation, agriculture, farmers, extension service, social support, food security, health, perception, West Africa

## Abstract

In West Africa, climate change aggravates subsistence farmers’ vulnerability to weather variability to sustain their agricultural and nutritional requirements. For successful adaptation policies, in-depth understanding of farmers’ perceptions about climate change, agriculture, and adaptation strategies is essential. This qualitative study in rural Burkina Faso characterized farmers’ perceptions and knowledge through in-depth interviews. The study enumerated the barriers, possibilities, strategies/practices, and support sources of farmers. There was awareness but limited understanding of climate change amongst farmers. Those unable to adapt, faced increased health difficulties, specifically regarding nutrition and mental health. Farmers could implement some dietary and agricultural adaptation strategies (reduce meal size, frequency and variety, preemptive purchase of cereals, multi-cropping, crop rotation, modified seeds) but were unable to implement others (soil rehabilitation, water management). Barriers to implementation comprised financial and time constraints, material and labor shortages, and inaccessible information. Farmers did not understand, trust or utilize meteorological services, but appreciated and relied on agricultural extension services. They reported that social and governmental support was sporadic and inconsistent. This study uncovers the following targets for climate change adaptation policies in rural Burkina Faso: promoting meteorological services, expanding agricultural extension services, increasing access to financial resources, and framing sustainable adaptation within national development goals.

## 1. Introduction

The Intergovernmental Panel on Climate Change (IPCC) stated that projected temperature increases in West Africa are “very likely” with mean annual temperature exceeding 2 °C above the late 20th-century baseline [1]. Climate change (CC) models project an additional two to three degrees warming in Burkina Faso by 2100 in comparison to 1990–2000 average [2]. On top of increased temperatures in the country, there will be changes in climate variability, more mini-droughts, heatwaves, torrential rainfalls and extreme weather events are likely [1,3]. Climate variability defined as “…*variations in the mean state and other statistics (such as the occurrence of extremes…) of the climate on all spatial and temporal scales beyond that of individual weather events*”, constitutes one facet of perceived and experienced climate change for populations [4]. These events are projected to have a negative impact on the crop yield of farmers in Burkina Faso [5]. Farmers’ will also face a plethora of direct and indirect CC effects. One such effect is reduced physical work productivity due to heat, heat stress and increased temperatures [6,7]. An indirect effect is that of the food and nutritional status of households, for which to this date, there are few studies Mank et al. [8]. Karst, et al. [5], Belesova, et al. [9,10], have studied crop yields, rainfall and temperature patterns and childhood malnutrition in the study area, the Kossi province [5,9]. Belesova et al., linked child malnutrition to trends in rainfall and temperature in the study area and projected child deaths from malnutrition till 2100 using climate impact models which reflect a 1.5 warming by the end of the century [10]. These studies indicate the importance of agricultural adaptation for the sustainability of subsistence farming populations [5,9,10].

Agriculture is at the intersection of CC adaptation to secure food and healthy nutrition in sub-Saharan Africa [11,12]. The agricultural/livestock sector is consistently ranked highest amongst the most vulnerable sectors to a changing climate, requiring sustainable adaptation strategies [13]. CC is and will continue to affect national agricultural productivity in multiple ways, most notably negatively, e.g., through reduced yield and affected crop quality [14,15,16,17].

Subsistence farmers in Burkina rely heavily on a single rainy season for crop production and income generation, in addition to food security and dietary adequacy of their household [18,19]. The sales of crops are essential for expenditure such as education and healthcare, which become inaccessible and unaffordable to rural households during a bad harvest season [20,21]. Small-scale farmers will be hardest impacted by the rapid changes brought on by CC [22,23].

The majority of these small-scale farmers in Burkina have a low ability and capacities for adaptation [24]. The Nouna area in the Kossi province was chosen because the population is particularly at risk of climate change impacts on health: they are highly exposed (depend on small-scale subsistence farming), severely vulnerable (low adaptatively capacity), and facing climate change hazards (mini droughts, rainfall variability) [1,2,3]. Adaptation in this study is defined according to the IPCC as “*The process of adjustment to actual or expected climate change and its effects. In human systems, adaptation seeks to moderate or avoid harm or exploit beneficial opportunities*” [25]. Without proper adaptation strategies, defined as “a *general plan of action for addressing the impacts of climate change… and measures that have the overarching objective of reducing vulnerability to climate change impacts…*”, [26] such households will be unable to sustain their agriculture-based livelihood and will continue to suffer from seasonal food insecurity [18,27]. These farmers’ understanding of CC, its effects on their farming and the required adaptation practices and strategies can make the difference between surviving short term versus sustaining for generations. Therefore, it is of the utmost importance to understand subsistence farmers’ perception and climate change and health. Furthermore, it is essential to determine their strategies of dietary and agricultural adaptation, along with the facilitators and barriers faced on their path to adaptation.

Through qualitative directed content analysis of in-depth interviews, the study aimed to determine the interrelations between the perception of CC among farmers living in North-Western Burkina Faso, their agricultural practices, and their strategies for adaptation. The specific objectives of the study were: (1) Characterize farmers’ perception of CC and CC impact on their health, (2) Enumerate farmer’s agricultural adaptation strategies (barriers and possibilities) (3) Identify farmers dietary adaptation practices (4) Determine farmer’s sources of support (social and governmental).

## 2. Materials and Methods

### 2.1. Setting, Population and Sampling Strategy

Burkina Faso is dominated by small-scale farming and subsistence agriculture, employing over 80% of the country’s workforce according to the Food and Agricultural Organization of The United Nations (FAO, 2014). The majority of small-scale farms range from three to six hectares on average and cover about one third of the national territory [28]. Subsistence farming is characterized here by rainfed agriculture, intensively cultivated by manual labor and in part assisted by livestock [29]. The Kossi province cites approximately 5% female subsistence farmers as heads of the household, but it’s important to note that majority of these, women practice an array of additional activities for revenue generation [30]. The primary constraints are poor water and soil management, and lack of agricultural inputs (fertilizer, herbicides, pesticides) and human workforce [31]. The country’s main staple food crops are sorghum, millet, maize, rice and fonio [32]. 

Although there are varying levels of uncertainty in climate modeling, a study by Boubacar, I., et al., 2014 illustrated robust features on the impact of climate change on rainfall in the West Africa region [33]. With the reference years of 1971–2000 and projections for 2021–2050 through 5 regional climate models, the authors showed a delay in the start of the rainy seasons by an average of a week and a consensuses on the lengthening of dry spells by approximately 20% [33]. Furthermore, the study showed an expected 3% decrease in rainfall events (0.1–5 mm/d) and a 15% average increase in strong rainfall events (50 mm/d) [33]. The increases in dry spell length will likely constitute a challenge for the agricultural system throughout Burkina Faso [33,34].

The Nouna area was selected for this study as it’s in a region affected by seasonal food insecurity, meaning that the population is under pressure to adapt their agricultural practices and diets to external factors [35]. The area is characterized by a high burden of child undernutrition, a single rainy season, and annual rainfall of approximately 800 mm [36,37]. Furthermore, the study site was selected because it’s population is heavily reliant on subsistence farming for their income and diets [36]. The Nouna Health and Demographic Surveillance System (HDSS) where our study was conducted covered 59 villages and counted approximately 104,674 individuals and 14,472 households in 2016 [38,39].

Thirty-two villages clustered around 5 weather stations were identified from the Nouna HDSS. From the above, 18 villages were randomly selected to be included in the study and thirty-two farmers were purposively selected from the villages. Purposive sampling is a method used widely in qualitative research to identify information rich cases and groups of individuals especially knowledgeable about the phenomenon of interest [40,41,42]. Participants were purposively selected for information richness according to the following inclusion criteria: permanent residency in the Nouna HDSS area, age ≥25 years, being the household head for at least one year, and subsistence farming being the primary occupation and source of income. Five selected farmers also acted as traditional regional chief, village chief, land chief or community spokesperson.

### 2.2. Data Collection

In-depth interviews were conducted using a semi-structured interview guide. The interview guide drew on existing literature about CC, health and farmer’s adaptation [43,44,45]. The interview guide for the study participants was edited and refined with input from the key informants. The guide was structured in four sections: (1) Introduction, (2) Perception, (3) Adaptation, and (4) Support Networks.

The interview guide was tested and adjusted prior to data collection, through four pilot interviews in Nouna with farmers (excluded from the main study) over the course of a workshop with a four-person team. The interviews were conducted, either at the participants home or place of work, in French or Dioula (local lingua-franca spoken almost universally in the study area) in October and November 2018 through open ended questions followed by probes and prompts.

Twice a week, debrief meetings were conducted amongst team members (principal investigator, local qualitative expert, trained assistants) along with memo writing. This was essential to check completed interviews, evaluate work progress and reflect on the data thus far. With this perspective over the work the principle investigator determined how much further data collection should continue. This was the assessment process utilized to begin highlighting main themes, adapt the interview guidelines and determine saturation.

The study protocol was reviewed and approved by the Ethical Committee of Heidelberg University (Identification Number: S-594/2018) and by the Ethical Committee of the Centre de Recherche en Santé de Nouna (CRSN) (Deliberation Number N2018-013/CIE-CRSN). All participants gave informed verbal and written consent to participate in the interview and to be tape-recorded.

### 2.3. Data Analysis

The recorded interviews were assigned anonymous identification codes, translated and transcribed verbatim in French and imported into Nvivo 12. First preliminary codes were created from reading the transcripts [46,47,48]. The preliminary codes were tested and amended by one additional researcher familiar with the study context and research subject. Next provisional coding was conducted [48]. The coded interviews were analyzed using a directed content analysis approach [49]. Categories were created and further developed into themes then linked to overarching concepts [50,51]. To reduce bias and increase credibility and validity, investigator triangulation was carried out with co-authors familiar with the study site and context, providing multiple perspectives and observations in the data, findings and the conclusions [52,53].

In presenting the results, the quotes were selected for poignancy and or representativeness of the research findings [54,55]. Each quote displayed in the results is identified with the participants’ study identification, occupation and age.

## 3. Results

### 3.1. Study Population

**Figure 1:**
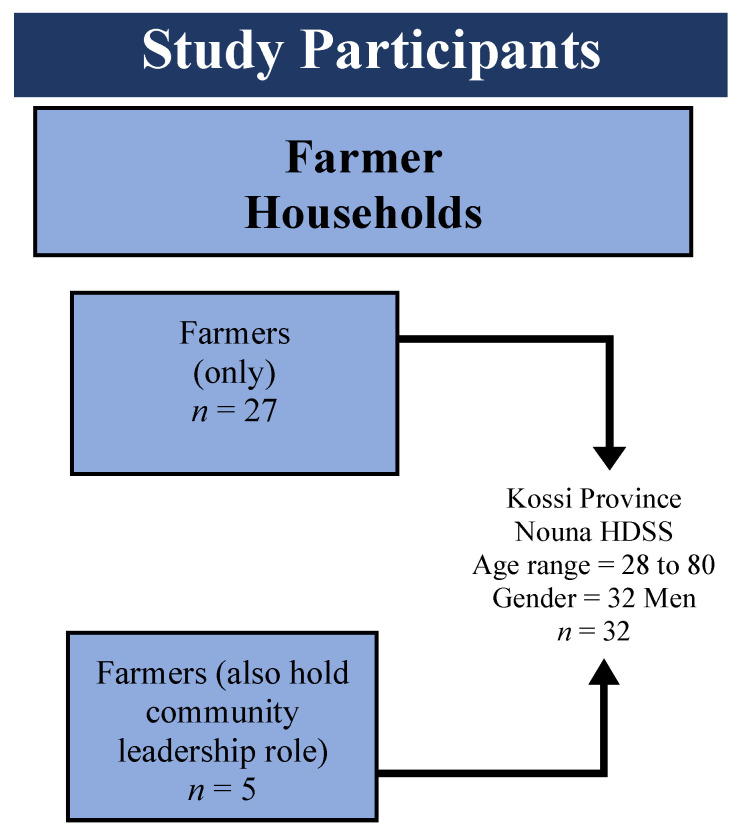
Description of the study participants in the Nouna Health and Demographic Surveillance System (HDSS)

Figure 1 presents the characteristics of the 32 farmers, 28 to 80 years of age, and all male.

### 3.2. Farmers’ Perceptions of Climate Change and Health 

#### 3.2.1. Perceptions of CC

##### Concept and Causes

Farmers used the words “changement climatique” in French or “wakati/wagati yêlêma” in Dioula to express their first encounter with the concept of CC, followed by their understanding of its causes, their related experiences and their feelings about the changing climate. All farmers, except one, were introduced to CC through one of four sources: local radio show, informal discussions, provincial service agents, and their own experiences.

When asked “what is climate change” participants described (1) changes in their environment which occurred over several generations and (2) rapid unexpected meteorological and precipitation changes experienced now and in the recent past. One participant stated that climate change means he is no longer in an environment “*like that of his grandparents.* (Climate Change) *are changes bit by bit along the time. The environment we grew up to find, is now over,* (no longer exists)” (25SRE_villagechief, 80). Participants described the biosphere/environmental change as slow changes over decade long periods of time, effecting heat, droughts, dryness, deforestation and desertification conditions of the environment. Participants also described their understanding of climate change as a change in precipitation patterns and timing “*…before it was raining a lot and on time, but now in the recent past, it* [rains] *only between June and July. This shows that there is a change*” (16KMD2_farmer, 30). Participants evoked unusual frequencies in meteorological events such as heatwaves and wind/sand storms, along with disturbances in the expected regions rain pattern and timeline.

In discussing the causes of CC, the farmers’ responses aligned under two categories: CC is due to “Godly Actions” and/ or “Human Actions”.

Farmers who thought of CC as a Godly action understood the phenomenon as a punishment due to human deviations from the general will of God (disobedience and hard heartedness), or communal failure to uphold the traditional lifestyle (unfulfillment of essential rituals and customs).

“[Climate change occurs] *because human beings have a hard heart. What God says to stop, we are not going to stop. If things do not please God, he will decide to punish us and if this punishment is not death, it will be the lack of rain or years with excess of rain, and our crops spoil. All this based on our work/actions. It is us who are not good, because there is too much bad faith/ill will among us. Meanwhile God is telling us not to be of bad faith, therefore the punishments bring about waste*” (25SRE_villagechief, 80)

Participants who pointed to human actions as the cause of climate change pin-pointed this in combination with nature-related actions. The abusive cutting of trees was cited as the primary problem. This was perceived to be due to growing village populations and an increased need for farming land, which leads to wood cutting and forest burning, resulting in desertification and droughts.

“*The causes* (of climate change) *are enormous. First of all, Man is the principle actor. With the increase in population, the needs of Man have become manifold. Because without money nothing can work. Whereas to have money you have to cultivate. To cultivate, you must destroy the forest, burn the trees and finally use the land as a field. Also, the machines, by the gases which they produce pollute the environment*” (27SIN_farmer, 42).

Seven out of the 32 farmers were unable to give a reason for the changing climate, stating that they have no understanding of reasons why their environment is changing. Each of those participants linked their lack of understanding to a lack of formal education *“… with the changes* (between) *before and now things are not the same, the impact it has, one cannot understand unless they were educated* (7DMD_farmer, 45)”. Participants explained that their lack of formal schooling leaves them not fully understanding the impacts of climate change. These notions of lack of understanding translated into a feeling of helplessness.

The farmers conveyed negative feelings about the changing climate and their current situation. They expressed discouragement, sadness, misery, weakness, and especially, fear with regards to their livelihoods, the food security of their households and their overall future.

“*One can say that it* [climate change] *scares us. Because in case of drought for example, we will ask ourselves the question “how to feed the family?” As with flooding, it is the same emotion of fear because we are heads of households. We will always ask ourselves the question of how to feed the family. So, it’s an emotion of fear that we have*” (27SIN_farmer, 42)

##### Traditional Weather Signs

Regardless of their understanding of CC, participants noticed changes in an array of signs, which they monitor as indicators of the changes in seasons and weather. These traditional signs comprise bird behaviors (nesting and passage), clouds and wind (rainclouds, cloud movement, density and color), and trees (flowering, leaf shedding, fruit producing) and more. For some households these signs also guide prayers and sacrifices to ensure a favorable rainy season. They are used across most households and are especially essential in households where members have not received formal education and/or do not use other knowledge channels for weather forecasts. These signs constitute key knowledge passed down through generations. One participant recounts the traditional signs he knows and uses *“…there are certain trees like the grape vine, when it makes flowers, then fruits, and the fruits start to ripen, we know that it is the beginning of the rainy season. It is by these kinds of trees that we make observations. As long as they are not ripe, we do not sow, as soon as the birds start to eat them, we know that they are ripe”* (22NNA2_landchief, 58).

CC is disrupting these traditional and natural signs. Participants expressed being disoriented about the start of seasons as many of the signs they relied on are no longer dependable or in some cases have ceased all-together. *“For us illiterates, the signs that we observe are in relation to the arrival of a bird with white wings called the Koli-Koli. They come to build their nests and we say that the rainy season is near. They come for a month, then they build their nests on the trees and then the rain will not take long to start to fall. That’s how it was before, but now they don’t come anymore”* (10DBL_farmer, 51).

##### Modern Weather Services

Weather services, which provide meteorological information and forecasts about rains, droughts, temperatures and wind; were not extensively utilized in this region. About half of farmers did not receive or rely on any modern meteorological information source. 18/32 farmers expressed receiving some form of weather forecast primarily through local radio show and informal discussions in the community and in farmer groups. *“We get this (meteorological) information sometimes. Even if you can’t hear it (on the radio), you can still hear it during community discussions”* (15KMD1_farmer, 55). Overall, only 5/32 farmers stated consistently receiving and utilizing information from the weather services for agricultural decision making.

The barrier to using meteorological information, as explained by farmers includes: lack of understanding of the information communicated; irregular access to the information; lastly, little trust in the accuracy of the information disseminated by the meteorological service. *“We listen to [the weather forecast] but not all the time. Sometimes the weather forecast, what it says is real/true, sometimes the information is not correct, sometimes it’s not dependable… we don’t use [the weather forecast] so much, since we use nature-based signs…”* (4BRS_regionalchief, 66).

##### Farmers with Leadership Roles

The farmers with leadership roles (*n* = 5) in the community were between 58 and 80 years of age, while the others (*n* = 27) were between 28 and 77 years of age. The leaders had a longer overview of the environmental changes in their localities, discussing 20, 30 and 40 years of changes.

Overall, their understanding of climate change and its causes aligned with those of the other farmers. Whether the community leaders cited “Godly Action” or “Human Action” as the cause of CC all their responses associated the current situation with disregard of social customs/traditions and the cutting of trees *“in the past, frankly said, there were a lot of trees, people respected the customs, and all that really made it rain enough. But nowadays there is no respect of customs/traditions and we have cut the trees…”* (4BRS_regionalchief,66).

The farmers with leadership roles used traditional signs in the same way as their counterparts and elaborated the same barriers in regards to the use of modern weather services. One leader exemplified the combined use of tradition and modern sources of information. To the question “Do you use any meteorological weather information or data?” He responded “*yes, absolutely*” elaborating that “*you have to use (meteorological weather information] to decide on your techniques. Use it to know [humidity and dryness levels), to know which crop is successful in relation to such humidity, to such drought…*” He continued then by explaining that at the start of the rainy season as a customary household “*We take out our masks either in May or in June or in July. If during the three or four days (of the customary ceremony) it doesn’t rain, it is certain that there will be something of a problem (in the season). But if it rains, (during the days of the ceremony) that’s already a positive sign (for the season)*” lastly to confirm the above he pays attention to traditional signs connected to bird migration, grasshopper appearances and caterpillars *“There are caterpillars, not the lesion caterpillars, small caterpillars red in color that appear at one point. If you see them appear, that also means that the season will be good overall. They are caterpillars that indicate good rainfall…”* (21NNA1_villagechief, 66).

#### 3.2.2. Perceptions of CC Related Agricultural Difficulties

In terms of land-related difficulties, farmers stated decreasing soil fertility, productivity, and nutrient content. In addition, they cited chemical soil disbalance as an equally concerning problem. Weather variability with regard to rainfall was deemed problematic by participants due to uncertainty and insufficiency during the rainy season. In addition to facing a cascade of natural events, flooding, bush fires, high winds and excessive heat, farmers’ fields were also overtaken by weeds, pests and diseases such as *Striga*, a root-parasitic plant.

Farmers perceived an increase in village and population size around farmed land, which has encouraged deforestation (cutting and burning trees, bushes, …) to create additional farming land. Farmers also stated facing none or limited access to farming equipment (pull type cultivator, plough, hoe, …) and agricultural inputs (organic and inorganic fertilizer, pesticides, herbicides). The loss of farm animals due to sale (as a result of financial constraints) or death has been unsettling for household for a multitude of reason. The animals provide effective strength to prepare the field, which increases the work efficiency and reduces work time per field. This loss of valuable time is compounded according to farmers by the brevity of the rainy season during which they can successfully plant.

Lastly, some of the farmers do not own or have legal right to the land they farm. One participant cited land ownership as the biggest problem in the past 5 years. Four other farmers indicted that the absence of ownership of land limits their ability to make long term decisions about their fields. This reduced their willingness to invest in time consuming, labor intensive or financially expensive field preparation and management.

The above-mentioned difficulties effectively resulted in reduced yield and thus less food and income for the household according to farmers. Farmers expressed that they had less core income to purchase additional food items and less disposable income for investments: *“What drought has brought me is misery. The money that I was supposed to take to pay for the children’s education I took to buy food. Imagine selling the oxen we use to plow [to make ends meet]. All that due to the drought”* (28TSI_farmer, 49).

#### 3.2.3. Perceptions of CC Related Health and Illness

##### General Health Status

Farmers had mixed perceptions on the development of health and illnesses in relation to climate change. A fraction of the farmers described a greater number of deaths in the past due to illness, in contrast to today. But the majority of farmers thought that there was an increase in incidence of illnesses today compared to the past 20 years. The majority also explained that there are new illnesses today which they were not previously aware of.

A recurring subject was the financial burden of health care, especially following poor-harvests, leading to catastrophic spending for households. *“…If you have no money from your agriculture, it’s from the millet which is here (that you will draw). (If) a child becomes sick, or for a baptism, or for the schooling fees of the children, you draw from the millet in your attic to settle these things. That truly is a great difficulty.”* (1BBK_farmer, 73). When they were not drawing directly from food stock to pay for care, farmers described their monetary saving being decimated. *“If we earn 10,000 or 15,000-Franc CFA (equivalent to 17-26 US$), we save. We pray to God that someone will not get sick. If only one person is sick, the money that we have saved, it is from there that we take (money) to go treat the person. If we go to the health centers, by the time we return, we come back empty-handed*” (18KROS_farmer, 51).

##### Nutritional Status

Farmers were acutely aware of the relationship between household nutritional status, stress, the occurrence of illness, loss of labor for farming, and its cyclical effect on the household. *“(If) you are not eating well, you are stressed. Stress, poor nutrition is already a big disease. Because you are stressed, (wondering) “will I be able to provide cereal grains for my family tomorrow?” it plays on the physical and mental health of the farmer”* (21NNA1_villagechief, 66). He continued by explaining that the stress levels of such situation are increased when a member of the family is ill. *“Now when the child gets malaria it is difficult, you cannot even eat, where will you get the money to go to the CSPS (local health post). Even if the state says it’s free, it’s for a minority. It’s (free) for children of a certain age, not for everyone. A teenager who is 18 years old, he is not taken into account…We can’t face that”*.

Household auto-sufficiency and the threat of hunger were a constant worry for all of the interviewed household heads who carried the responsibility of providing food for their family. They expressed constant stress, worry and anxiety, affecting their physical and mental health.

### 3.3. Farmers’ Agricultural Adaptation Strategies

#### 3.3.1. Possibilities for Agricultural Adaptation

**Figure 2:**
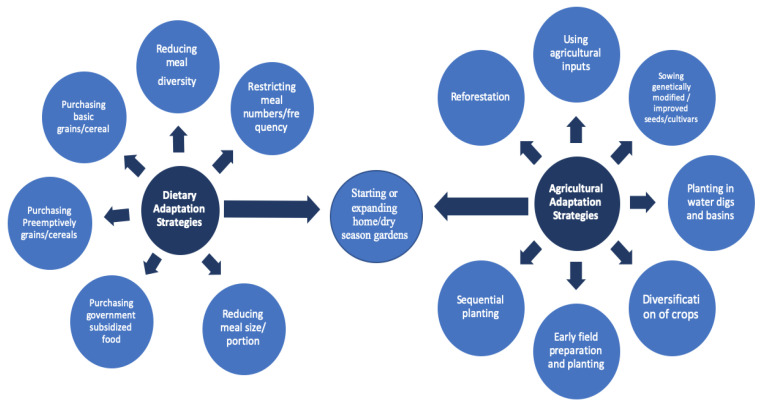
Reported dietary and agricultural adaptation strategies as stated by households.

To brace themselves against the effects and repercussions of the changing climate, every participating household has recently made changes to their farming methods. In addition to methods already widely used in the region, such as multi-cropping, crop rotation and the use of organic fertilizers, farmers reported having adopted an array of new methods in the last 3–5 years in order to increase their chances of a successful sowing season and to maximize their harvest outputs. The methods employed by farmers include: the use of agricultural inputs (organic and inorganic), sowing genetically modified or so-called improved seeds and cultivars (when available), setting up of home/dry season gardens, diversifying crops, early field preparation and planting (before the start of the rain), sequential planting (plating again after failed first round), planting in water ditches and basins, and lastly reforesting (Figure 2).

One participant, who has been farming in the region for over 30 years, described a string of changes farming has undergone *“… before, for thirty years, it was only the plow, then they brought the powder to mix with the seed before sowing. We found it weird because it exterminates all wild living things. Then they brought in the herbicides to kill the green grasses, the soil becomes very heavy and there is also fertilizer that brings about a change… there are really a lot of changes”* (1BBK_farmer, 73)

With regard to the use of genetically modified or improved seeds and cultivars, there are now a selection of seeds available with varying characteristics. In this region, the most important and valued characteristic being a short maturation cycle, which allows the farmers to adapt to a shorter rainy season. *“There are several kinds of seeds, currently, there is a type of millet which has a very short cycle, that’s what must be used nowadays, whether the rainfall is sufficient or not, you will harvest a little. This too is a strategy that must be recorded and put into practice. It’s obligatory today to use them”* (1BBK_farmer, 73).

In addition to these new adaptation strategies, the participants discussed adaptation methods that they were not able to implement on their own fields. Study participants explained that they heard of these methods through the provincial services (more specifically technical agents from the agricultural office) or saw them in other villages. These include the soil and water management strategies called zai, half-moons and stone contour/lines (Table 1). *“The technical agents of the agriculture extension service have very well explained to us (farmers), that when the time comes, if the water from your field flows downward, you go to line stones like a barrier and if you can, cover it with earth. And if God causes the rain to come, the water does not pass, the water stops to humidify the interior (of the field). We even went to see. If you go to the Djibasso department, the people there do that. But it is not done here yet”* (25SRE, Village chief, 80).

In describing the methods, they could not use in their fields, farmers were highlighting adaptation barriers. “*What I know and I can’t do, is because I don’t have the equipment and if your field is very large and you don’t have enough labor, that’s also a difficulty, because each material has its task. You know, you are aware of a task but you lack the means to do it, this delays your work*” (30TNI2_farmer, 44).

#### 3.3.2. Barriers to Agricultural Adaptation

The enumerated barriers to adaptation were mainly linked to the lack of resources. They can be categorized as follows: finances and loans, information (education, government resources), labor, farmer organizations, farmable land, time, and transportation (Table 2). The participants reported facing combinations of these barriers simultaneously with little hope of finding solutions.

### 3.4. Farmers’ Dietary Adaptation Practices

In addition to changing their agricultural strategies, farmers and their household also changed their dietary practices (Figure 2). Here dietary adaptation practices are defined as actions or behaviors by households to manage their food intakes and meal structures throughout the year [59,60]. Over the past 3–5 years households have utilized the following practices: purchasing basic grains/cereals, establishing or expanding home/dry season gardens, restricting the number of meals, reducing the diversity of meals, reducing the quantity of meals, purchasing government subsidized food (when available), and preemptively purchasing grains and cereals (Table 3).

As households increasingly face the uncertainty of food production and food security, they reported additional difficulties surrounding food consumption and acquisition: (a) The increasing prices of grains and cereals on the market, due to the higher demand in the village as more households turn to the market to secure sufficient grain/cereals for the entire year (b) Growing concerns about consuming crops filled with unknown and dangerous chemical products and growing crops with origins and nutritional contents they are unaware of (c) Spending part of households cooking budget on purchasing wood for open fire cooking, as the wood can no longer be easily collected in nearby bushes or forests, due to increased deforestation to create farming land. In an effort to minimize the purchasing of cooking wood, households stated utilizing/repurposing farming and animal residues. Households have in the past used animal residue as fertilizer on their fields, but today they use every part of their farming and food residue to feed animals and burn it as cooking wood/fuel. *“They (the women) collect the embers to make charcoal. Whereas before that was not the case… and then the grains, (remaining after making dolo). Before we threw it away, but today it’s sold. People feed cattle with it*” (21NNA1_villagechief, 66).

### 3.5. Farmers’ Sources of Support

#### 3.5.1. Social Support

The enumerated sources of support (social and financial) for farmers in the Nouna HDSS area were (1) family and friends living within the community, (2) family and friends living outside the community (at mining sites, in Ivory Coast, in Europe), (3) government services (social, agricultural, forestry services) and (4) Non-Governmental Organizations (NGOs). Of these four sources of support, not one has been described as a constant source of support.

The support ranges from sporadic one-time aide money transfers by family members working within the West African region—“*We received help from a person* (outside the household). *We had a child who was in Ivory Coast helping us but he died this year*” (17KRO1_farmer, 60)—to sporadic money transfers from multiple family members in Burkina Faso and in Europe—*“There is a* (family member working) *in the mines and another abroad in the white man’s land, often they send us money to pay for food”* (20NKI2_farmer, 60).

Farmers also received nutritional and academic support for school-aged children from NGO’s and social services. “*Last year they* (the NGO social workers) *called me, saying that they are going to help for* (the school fees of) *the children. This year the school said that if the kids don’t pay the money* (for the school fees), *they can’t go to school. They* (the NGO social workers) *have helped last year but not yet this year…I haven’t heard anything yet from them*” (18KRO2_farmer, 51). Like the family-based support, there was no guarantee of consistency from the NGO and/ or social services.

#### 3.5.2. Governmental Support

The sporadic nature of help received is further described in association with the agricultural extension services. Households portrayed vastly different levels of interaction reaching from once every couple of years during community counseling sessions, to multiple times within the same agricultural season, during various community counseling sessions, group methods demonstrations, lending of agricultural equipment and even individualized visits to specific fields.

Although contact between farmers and agricultural extension service agents varied, every single interaction was reported and recounted positively by farmers. There is a high level of satisfaction with various services provided by the agricultural extension agents. Additionally, all participating farmers had a high level of trust in the agents and the information shared by them. *“The agricultural agents yes, I trust them because the pits that we dug, the dimensions were given by them. In addition, they gave us the materials to dig and collect, such as shovels and wheelbarrows. Also, they gave plows to some as well as seeds of sesame, corn and others. Some even got oxen; others also fertilizer”* (27SIN_farmer, 42).

This trust was based on two reasons (1) Farmers displayed a high level of appreciation for the efforts extension service agents made to reach their village and to accompany their agricultural activities and (2) Farmers unanimously agreed that the advice of the agents led to positive results and improved harvests. *“They (the agricultural extension agents) come at the beginning of the season, at the sowing time. They ask for the number of hectares and give advice, on the amount of inputs or manure you need to put. As he drives by, if he sees a field, he asks, who owns this field and he also gives advice”* (26SBN_farmer, 45). The farmers found it unfortunate that they do not receive more visits from the agents *“…they (the agricultural extension agents) don’t come often anymore… In the past five years they might pass by not more than once a month, it looks like they are discouraged. They go into the village to inquire and they continue on their way. Once, I asked one of them ‘why are you not coming over?’ They said they are no longer given (motorbike) fuel like before, so they can’t do a lot of touring anymore”* (26SBN_farmer, 45).

Participant 4BRS, a regional chief and currently a full-time farmer, who retired as a field agent from the agricultural extension service explained, *“Well, where it is really a little regrettable is that the agents are no longer numerous, and they do not have the means to travel. That’s what’s a little bit unfortunate. Well in the past, the agent had a few villages and he toured many fields. At the moment (the provincial office) doesn’t give enough operating funds. If there is not enough money, then for an entire area, you will find one or two agents, who have to often cover countless producers. It’s like taking a packet of sugar and throwing it in a well”* (4BRS_regionalchief, 66).

In comparison to the past twenty years, the frequency of visits have decreased in farmers opinion as there are fewer agents covering increasing superficies/villages and as agents receive less financial support from the government for their field excursions, specifically payment for transportation, travel and field visits.

## 4. Discussion

### 4.1. Summary of Main Findings

This study determined the interrelations between the perception of CC among subsistence farmers living in northwestern Burkina Faso, their agricultural practices, and their possibilities for CC adaptation. More specifically; we characterized farmers’ perception of CC and health, enumerated farmer’s agricultural adaptation strategies, identified household’s dietary adaptation practices and determined the most important sources of support for farmers—information which is key for utilizing bottom-up approaches to build adaptation programs geared at assisting the respective population.

The study results indicated that nearly all farmers were familiar with the concept and consequences of CC, believing that it is caused either by Godly actions or human actions. Households faced barriers in their efforts to adapt, related to financial difficulties, time constraints, materials and labor shortages, inaccessibility of information and support (formal and informal). Farmers enumerated adaptation methods, which they were able to use at varying degrees: multi-cropping, crop rotation, fertilizers, genetically modified seeds/cultivars, home/dry season gardening, planting diversification, early field preparation/planting, sequential planting, planting in digs and basins, and reforesting. Methods they were unable to use included the zai, half-moons and stone contour. The farmers linked CC to health through mental health and food security. Famers adapted their dietary patterns by reducing or restricting the quantity, frequency and diversity of meals, purchasing grains/cereals and purchasing government subsidized food. The participant’s sources of support were family and friends living within and outside their community, NGOs and provincial government services. Lastly farmers did not see the weather service as an accessible and alternative source of meteorological information but showed trust and confidence in the agricultural extension service.

### 4.2. Perceptions of CC and CC Impact on Their Health Health

The farmers in this study displayed a high level of awareness of CC and its effects on their livelihoods and environment. This is in line with a number of studies among West African farmers, which reported increasing levels of CC awareness [24,43,61]. In our study region of northwestern Burkina, as well as in a publication with data from the central plateau and the southwest region, farmers have expressed decreasing ability to read the local, traditional, and natural signs for weather and seasonal change due to CC-related developments [62]. This is creating an information gap which could be filled with science-based weather information as it’s essential for farmers to have meteorological information when making agricultural decisions [63]. The combination of traditional and scientific knowledge is possible as Roncoli et al. showed that farmers do not think of these two sources of information as self-exclusive, but instead have displayed the capacity to use multiple cognitive frameworks to examine weather. With support, farmers could incorporate both traditional local and modern scientific weather information into their decision-making frame [63]. This would help farmers bridge the knowledge and understanding gap between traditional and modern weather forecasting [64,65]. To make this possible, it is crucial that (a) reliable localized meteorological information is available to remote and illiterate farmers, (b) farmers receive regular access to weather information through locally available radios or other local communication means, and (c) farmers are led through the integration of science-based weather forecast into their agricultural decisions in a participatory manor. A 2009 study in Northern Burkina Faso has shown that forecast dissemination, through participatory workshops can be an effective first step in transitioning through the incorporation of scientific information in farmer decision making processes [66]. Participatory dissemination workshops can play a positive role in the provision of effective climate services to African rural producers, in their understanding of the data and their utilization of the information [67]. Furthermore, this participatory workshop dissemination approach can lower the hurtle of integration by addressing two of the barriers to farmers utilization of scientific information: lack of understanding and low trust in the information. Importantly, these barriers are intertwined—compounding the difficulty—as part of the mistrust is lack of understanding and inability to effectively translate the information into action [62,64,66]. Such participatory workshops could potentially also be utilized in addresses rural community and farmers CC-related health concerns.

The relationship between CC and human health for the west African farmer in this study was enumerated through food security and mental health [68,69]. Although these represent two well know CC-to-health-pathways [70,71,72], limited literature is available on the effect of these pathways on the rural farming populations, especially in West Africa [19,73,74]. In addition to the nutrition and mental health pathways, the literature has established a number of other health effects related to climate change. Haines and Ebi illustrate health pathways through four CC events (rising temperatures, extreme weather events, carbon dioxide emissions and seas levels), which impact the social determinants of health (socioeconomic, demographic, environment). Those lead to heat stress, food supply shortage and safety deficits [71]. Some pathways, not enumerated by farmers but still relevant to them due to their lifestyle and livelihood, include injuries and fatalities in extreme weather events, cholera and bacterial diseases through low water quality and quantity, forced migration and physical violence [71]. This illustrates that farmers are vulnerable in a multitude of ways—some of which they are acutely aware and others they have yet to understand. Because CC adaptation is not only important for agricultural but for overall health it is imperative that further research be conducted on the effect of CC on farming populations. Furthermore, the CC to health pathways which result in ill health will trigger an array of socioeconomic consequences for the entire household, pushing it further into vulnerability [23,61].

### 4.3. Dietary Adaptation Practicies

The criteria for food security (availability, accessibility, utilization and stability), which exists when everyone in the household at all times has “*physical and economic access to sufficient, safe and nutritious food to meet their dietary needs and food preferences for an active and healthy life,*” are increasingly interlinked with CC and unattainable to subsistence farming households in this study and across sub-Saharan Africa [75,76,77]. For households in our study food availability was highly compromised by CC’s effect on domestic production and by the inconsistency of government subsidized food aid. Households access to food was compromised by their inability to access information on subsidized food distribution, their inability to access profitable marketplaces, and the unaffordability of cereals/grains based on their limited purchasing power. Utilization which relies on food safety, quality, nutritional knowledge and proper preparation is also threatened as households in our study expressed concerns over the consumption of potentially dangerous chemicals (herbicides, pesticides, inorganic fertilizer) applied to crops and the households reliance on new forms of genetically modified seeds/cultivars, whose origins and nutritional values are unknown to them [78]. The stability and maintenance of the above listed food security pillars was rendered even more difficult as households are turning to nutritionally detrimental and unsustainable practices for dietary adaptation [78].

The dietary adaptation practices utilized by households in our study primarily aimed to stretch food supplies overtime permitting the households to make it through to the next harvest. The adaptation strategies focused on minimizing food consumption (through restricting and reducing meal quantity, frequency, variety) or increasing food availability (through purchasing cereals/gains on the marketplace and purchasing government subsidized food when available). The strategies aimed at minimizing food consumption negatively impact the nutritional; status of the household resulting in food insecure households. The efforts to increase food availability, such as creating and expanding home/dry season gardens, employed by household in this study is supported by literature and encouraged by international organizations, such as the United Nation Food and Agricultural Organization (FAO) [79,80]. This is especially important for households threatened by climate-induced harvest or income losses. Home gardens are characterized by the production of a variety of legumes and vegetables on small plots of land inside or in proximity to family homes and compounds [80]. Agricultural field and crop yields constitute the majority of energy needed by households, while home gardens can supplement the household diet with vitamin-rich vegetables, herbs and condiments [79]. Home gardens, also known as kitchen gardens, when adapted to local, cultural and traditional preferences and agronomics are sustainable [80,81]. They can help households increase food quantity, increase dietary diversity and the intake of micronutrients [81], in addition to providing additional income for households through sale of unconsumed produce [80,81]. Lastly home gardens can be a source of available, accessible, usable and stable nutrients and nutrition, sustainably increasing the food security of the entire household [78,81].

### 4.4. Agricultural Adaptation Strategies

The barriers and difficulties faced by subsistence farmers today are interlinked with CC in one of two ways: (a) Pre-existing farming difficulties exacerbated by CC and (b) New farming barriers due to CC. 

Juana et al. demonstrated that subsistence farmers have historically faced difficulties with their finances from lacking the financial capital to purchase agricultural inputs, farming equipment and means of transportation, to lacking access to loans which facilitate the timely purchase of necessary materials. Today due to CC, a successful and sufficient yield is increasingly dependent on equipment and input. Pull type equipment is now essential to complete the sowing of fields. Organic and inorganic fertilizers are now essential to sustain and increase the nutritious qualities of depleted topsoils. The use of genetically modified and/or improved seeds/cultivars with short maturation cycles is crucial to increase harvest output even during short, disrupted rainy seasons [82]. The exacerbation of pre-existing difficulties through CC is also evident through the sale of farm animals. In West Africa, farm animals were always sold as a reactive adaptive strategy to offset household emergencies such as illnesses and funeral costs [83,84,85]. Farmers today are selling essential farming animals not only as a result of crisis but as an anticipatory adaptation strategy, resulting in a cascade of difficulties in the coming season due to the loss of labor from field animals that were sold [84,86].

Loss of labor is not just a secondary effect from the sale of animals. It is a new difficulty for farmers, household and communities due to CC induced migration [84,87]. In agricultural communities, young people are leaving behind farming and their villages in search of new sources of work and income [86,87]. Unlike seasonal migration for income/livelihood diversification, this migration is no longer restricted to the “dry season” It’s permanent and often seen as a last resort [84]. This pattern, not limited to Burkina Faso, has been recorded throughout West African and across the world [88,89,90,91]. Those who no longer view farming as a reliable source of subsistence have cited a plethora of reasons including changing rains, environmental degradation, and increasing extreme weather events [92]. In regards to land, farmers have cited lack of land ownership as a new barrier to adaptation. Even though legislation in 2007, 2009 and 2012 have aimed to formalize and legalize land ownership, many countries like Burkina Faso still have various customary ownership and traditional land tenure systems in place [93]. Farmers on traditional leasing systems are less likely to invest in labor or financially expensive adaptation methods [93,94,95]. Since they do not own the land, tenants have no guarantee of benefiting from their efforts, especially when results and rewards are not immediate [93].

### 4.5. Sources of Support

When facing farming difficulties farmers rely on their support systems. Their social networks, friendships, kinships, and social groups constitute a primary source of support, ranging from channels of knowledge sharing to sources of financial support [44,96]. Farmers also rely on government services, the most utilized of which was the agricultural extension service. Furthermore, as illustrated in our study, evidence across sub-Saharan Africa suggests that farmers appreciate advice from extension services, but services tend to be limited and population coverage declining [97]. One of the barriers to illustrating the direct benefit of extension services was the inability to quantitatively illustrate the positive effect extension services have on farmers. The primary difficulty was controlling for bias, as farmers, who sought agricultural extension services, were more likely to also be respectively better off financially. Owens, T. et al. has been able to control for it, illustrating that one or two extension visits per cropping season has a statistically significant impact on crop production [16]. More specifically, such visits raise the value of crop production per hectare of cropped area by 14.4 per cent [16].

The provincial government offices, covering forestry, agriculture and livestock/animal husbandry, unlike most nongovernmental organization and community-based organization in the province, are long established services—active since the 1960s—which will continue to be sustained in the future by the national government [98]. This makes them well situated to accompany households in long-term planning and adaptation to CC. With additional funding and improved structural approaches provincial services, especially the agricultural extension service can have a greater impact on the communities they serve. Additional funding can support the expansion of successful household-based programs like increased visits to farmers plots, and community-based programs such as Farmer Field Schools [99,100]. A structural approach at the provincial level could help translate policy to program and then translate program to practice. At the policy-to-program formulation level (between the nation ministry and provincial services) there is a top-down approach, but at the program-to-practice level (between provincial services and the population) there should be a bottom-up approach [100,101,102,103]. Reference [100] suggests a balance of the two approaches (top-down and bottom-up) can ensure that the policy translated to first to program then to practice, are aligned with national goals and are appropriate for local conditions. At the program-to-practice level, it’s important that extension services include farmers, and farmer groups and local stakeholders during the program to practice/implementation phase, as maximizing their involvement can increase overall acceptability at the population level [16,97,104].

### 4.6. Strength & Limitation

Farmers’ adaptation and ability to cope with weather impacts is not only dependent on environmental but also on socio-economic factors such as age, gender, education, income, assets, household size, farm size and farming experiences. [105]. The study by Ndamani and Watanabe indicated that household income, education, and household size influenced farmers’ adaptation to CC in Ghana. The subsistence farmers from the Nouna HDSS area were homogeneous in regards to education level, income, assets and farm size, thus these factors were not integrated in the sampling and analysis [5,106]. With regards to gender, the study targeted interviews with heads of households who are primarily men. Although women contribute to agricultural labor in households, within the Kossi province men remain the decision-makers in regards to agricultural planning, activities and changes [107,108,109]. They are also the household member with the largest overview of household harvest and crops [108]. Within the Nouna HDSS 59 villages and 1 town 8.19% of households are headed by women. In the 18 villages of this study 4.47% of household heads are women and a large part are not primarily subsistence farmers [110]. Though some female household heads farm they often practice an array of revenue generating activities including but not limited to the provision of domestic services, the sale of crafts and home brewed products such beer, which are primary sources of revenue for their households [109]. The non-inclusion of subsistence farming, women heads of households might bias our findings towards a neglect of female empowerment in all its forms as an integral aspect of climate change adaptation [109,111]. As a result, of the elimination of gender, education, income, assets and farm size—age and farming experience– the two influential socio-economic factors remaining, were used to draft the participant inclusion criteria. 

A strength of the study is the active reduction of researcher biases at various stages: (1) data collection, (2) data analysis, and (3) results presentation [54]. The reduction of researcher-introduced bias at these three stages along with ample information on the study participants and research context are key to facilitating transferability of the research [112].

1. At the data collection stage, the presence of the interviewer can have an undesirable effect on or create a barrier to open-up by the interviewee [107]. This was minimized in two ways: (1) the interviewed participants are part of a HDSS population accustomed to the presence of researcher in their community, and (2) the principle investigator and the accompanying field agent were all Burkina nationals, with years of experience in the Kossi province and in tune with the culture and customs of the population [113]. This minimized the “outsider effect,” facilitated contact with the study participants and allowed the study team to quickly establish a friendly and comfortable rapport [114]. Furthermore, the principle investigators’ high level of sociocultural competence and background knowledge contribute to ensuring the credibility and confirmability of data and findings [115].
2. At the analysis stage, translation of interviews can be a significant threat to the validity of qualitative research [116]. Poor translation can reduce richness of context and increase content deviations. To minimize the creation of distance between meaning as experienced by study participants and meaning interpreted in the results, interviews were conducted in French whenever possible [117]. The interviews conducted in Dioula were translated to French soon after the interview. The translation was conducted by well-trained local research staff, fluent in both languages and with a decade of experience in translation. Furthermore, the analysis was conducted in French, avoiding the translation of the entirety of the data set from French to English. Only the quotes utilized in this publication were translated to English. This translation was checked by two native speakers to retain the original meaning.
3. At the results stage, researchers must ensure that the findings presented are of quality and reflect the experiences of the participants. First, a results dissemination meeting with the study participants was conducted in Burkina Faso in early 2020. This meeting allowed participants to reflect on the findings, and express whether the findings adequately portrayed their experiences and the messages they conveyed during the interview. This feedback was integral to the results validation process, which was incorporated in the final draft of the paper. Second, the “Checklist for Qualitative Papers” as provided by Anderson 2010 was used to guide the presentation of results [54].

### 4.7. Perspectives for Researchers and Policy Makers

In conclusion the study makes the following recommendations in regards to research and policy. We identified a lack of qualitative research on the effects of adaptation mechanisms to climate change in subsistence farming environments and their impact on food security and health of households. Such research should assess demographic, socioeconomic and environmental factors influencing health of this vulnerable population and determine the resulting physical and mental health outcomes. This would serve to protect the health of farmers, central to their ability to adapt sustainably rather than on a yearly basis. Furthermore, research should explore the perception of relevant provincial service agents (employed in forestry, agriculture and livestock/animal husbandry) and stakeholders (domain experts, decision-makers and policy makers) by mapping their understanding of CC, adaptation, agriculture and health. This would serve as a frame for understanding the current policy direction of the nation in regards to CC adaptation and plans for implementation. Such research would provide information on potential knowledge gaps at the stakeholder level which can result in policy gaps while also highlighting ways research can better assist science-based decision making and policy writing.

Farmers across West Africa use a multitude of adaptation methods, many of which are poorly adapted for the long term. In our study farmers listed methods such as deforestation to create new farming land, the use of inorganic fertilizers, and unlicensed pesticides and herbicides, which are detrimental to farmers’ health and the soil especially when used incorrectly for extended periods [105]. Although such practices yield immediate results, they have negative repercussions, ranging from chemical imbalances in the soil to the contamination/pollution of water sources [118]. This highlights the fact that sustainability must be at the forefront of adaptation. Climate Smart Agriculture (CSA) embodies the sustainable direction West African countries should be taking in CC adaptation. As described by Zougmoré et al., 2016, CSA *“(1) sustainably increases agricultural productivity to support equitable increases in incomes, food security and development; (2) adapts and builds resilience to CC from the farm to national levels; and (3) develops opportunities to reduce greenhouse gas emissions from agriculture compared with past trends” [119]*. CSA promotes the use of methods such as the integrated soil fertility management, wetting and drying approach and agroforestry [57,118,120]. The adaptation strategies should be sustainable, which is why sustainable adaptation should be integrated in long term policy and development goals of a country [101].

The government can impact farmers’ adaptation by reinforcing extension services, especially in poor rural settings. This can be done by (1) expanding the coverage and services offered by extension services, (2) training extension service agents on CC, and (3) applying a bottom up-approach from policy to program, and from program to practice. Expanding the coverage of extension services requires increasing the financial resources allocated to these services [61]. This can be beneficial by allowing the offices to provide a larger array of services at a higher frequency. The reinforcement of the extension services should include specific CC training. With reinforced training on adaptation methods and access to poignant resources, such as the FAO Climate-Smart Agriculture: Training Manual for Agricultural Extension Agents in Kenya [56]. The agents will continue to be a trusted source of information and knowledge for farmers [97], sharing well-researched methods along with practical training [104].

Burkina Faso is deemed one of the most socially, infrastructurally and climactically vulnerable countries on the African continent [121]. Subsistence farmers are among the most vulnerable group in the country; whose poverty will deepen as the projected negative effects of CC increase, stress on water resources become permanent, the incidence of mini-droughts during the rainy season increase, yields decrease, food insecurity increase, and human health difficulties develop [122,123]. Where CC is regarded as a marginal issue, it will hamper the social, economic and sustainable development achievements of the country, if it is not integrated into national policies [124]. This is illustrated through the interconnectivity of the 2015 Sustainable Development Goals (SDG). Without Climate Action (SDG13) many developing countries will be unable to reach others SDG goals such as No Poverty (SDG1), Zero Hunger (SDG2), Good Health and Well-being (SDG3) [125]. CC and adaptation must be incorporated in the long-term planning at the national level, and aligned with the national development goals of the country, because socioeconomic development influences a population’s ability to understand and adapt to CC [102,124].

## 5. Conclusions

The study shows that farmers in rural Burkina Faso are aware of a changing climate and its impact on their livelihood (agriculture yield) and diet (nutrition), the knowledge and the ability to adapt without support. Financial, labor, equipment, informational/knowledge barriers to CC adaptation create a sense of fear and helplessness in farmers ability to sustain their lifestyle and food insecurity. The results of the study also highlights numerous possibilities for researchers and policy makers to assist farmers on their path to CC adaptation by (a) bridging the gap between traditional weather signs and modern meteorological information, (b) increasing farmer’s access to financial resources, (c) reinforcing government provincial services and (d) integrating CC adaptation in long-term policy and development goals at the national level ensuring sustainability and continuity.

## Figures and Tables

**Table 1:** Adaptation strategies discussed by farmers which they were unable to implement.

	Adaptation Strategies Not Implemented by Participants	Description of Strategies
1	Zai, micro pits or planting pockets	The zai is a soil rehabilitation management system in which organic matter is buried in small pockets/pits. This helps restoring soil fertility and conserving water in land/soil. The pits are shallow, wide, circular and hold both moisture and manure [56,57].
2	Half-moon or semi-circular bunds/basins	The half-moons is a water management system, in which soil embankments are created in a semi-circle form. They are of varying dimensions and work for rangeland rehabilitation, crop growth, shrub planting and also for fodder production [18,58].
3	Stone bunds/contour/lines	The stone bunds/contours are useful for harvesting, and slowing down and filtering rain and runoff water. They increase infiltration, capture sediments and reduce erosion. [18,58].

**Table 2:** Barriers to adaptation faced by households.

Agricultural Adaptation Barriers	Description of the Barrier from Codes	Participant Quotes
Finances	Lacking financial resources to invest in new/alternative farming methods: purchase farming machines & technology purchase agricultural inputs (fertilizer, herbicides…) hire farm human labor or rent animal labor	*“There is the lack of financial means, there are a lot of things we want to do but we cannot. If you cannot afford to buy fertilizers, for example, fertilizers are extremely expensive, you might have to put three bags in one hectare, but for lack of means you have to put maybe two bags or one bag…”* *(4BRS_regionalchief,66)*
Loan	Lacking access to formal loans due to: difficulty fulfilling requirements application difficulties and literacy barriers fear of inability to pay back loans in time fear of becoming further/indefinitely indebted	*“To get a bank loan you must have something yourself to deposit as a guarantee, if you don’t have that, you can’t get up just like that and get a loan, who will give you one. I never got one” (2BRK_farmer,56)*
Labor	Lacking physical strength due to age Lacking physical strength to work the land Lacking labor source in household Lacking labor source due to migration Lacking options to hired labor / animals	*“The work is very tiring and it requires a lot of labor while almost all our children are in school… we want to do the new practices, only it is the time, the lack of funding, and the workforce labor that creates a problem…” (27SIN_farmer,42)*
Materials	Lacking farming material: equipment: pull type cultivator… inputs: fertilizer, pesticides, herbicides	*“If you want to change your farming, the difficulties that you meet are numerous. There is the question of fertilizer, there is the question of plows, oxen, everything that is material. If you don’t have the work material and you want to change, how will that occur” (22NNA2_landchief,28)*
Farmer organizations	Lacking farmers organizations /associations to: conduct community initiatives conduct labor/work for/with farmers support one-another with information, ideas create access to formal financial loans	*“It is important, even if it is just to sit down talk and give each other ideas. Often, we borrow money from the association to go do what we want and pay back afterwards. So, in a group there are many advantages” (10DBL_farmer,51)*
Information. Formal Education	Lacking information/knowledge about alternative methods Lacking formal education Lacking access to written information	*“All this [difficulties listed] is due to illiteracy, otherwise when you are educated, and everyone has a little knowledge, it is not as difficult”* *(22NNA2_landcheif,58)*
Government	Lacking access to: to government programs and initiatives to politicians/government officials	*“If you have a relative in government, there is some assistance/aid you can get quickly. But if you don’t have someone in government, you hear about aid, but it goes somewhere else, it doesn’t come to you”* *(25SRE_villagechief,80)*
Farmable Land	Lacking resources/knowledge for land revitalization Lacking access to unfarmed/nutrient rich land Lacking ownership and legal rights to land	*“The land is no longer fertile and we have no more/ new land to cultivate. Since I started to farm, it is the same corner of land that we are cultivating still. We manage to put a little manure, otherwise there are no solutions” (42GNI3_farmer)*
Time	Difficulties aligning start of rains and sowing Increasing time constraints due to inconsistent rains Insufficient time to implement an adaptation strategy	*“When you have several fields, and you want to do this practice [stone contour], you will not finish in time since the rains end prematurely. So, you have to do it quickly, to catch the start of the rains early. If you want to plow and sow with the contour, by the time you finish, the rains will already become inconsistent, it [crops] won’t grow and the season ends, that’s why we don’t do this” (3BRO_farmer,43)*
Transport	Lacking means of transport: to seek help from extension / provincial service offices to investigate strategies in other villages for materials for adaptation strategies for agricultural products to reach storage facilities for agricultural products to be sold in best price markets	*“For example, stones [for the stone contour], if you don’t have a cart you can’t go pick up/transport them. If you implement it [the stone contour], its good. Before there were tree trunks that we had placed to block the water but all that is rotten now. To go to the field too, there is no cart” (20NKI2_farmer,60)*

**Table 3:** Dietary adaptation practices of households.

Dietary Adaptation Practices	Description of the Practices	Participant Quotes
Purchasing basic grains/cereal	Households that consumed the entirety of their harvest even before the end of the year, must then purchase the same grains/cereal on the market, which were historically entirely provided by their own farm yields.	*“There has been a change because before we didn’t buy grain, but for the past few years, before the end of the year, we have to buy grain to feed the family because what we produced was not enough” (6DMD1_farmer, 28)*
Starting/ expanding home/dry season gardens	Households that already had home gardens described expanding the variety of vegetables and strengthening their gardens to last throughout the dry season. Other households are for the first-time cultivating legumes in a home garden structure. Lastly, some households have seen the benefits of such gardens and are now hoping to implement one themselves.	*“… gardening was never discussed in the household; we didn’t have that in mind. But due to climate change, we have been collecting water and the children today produce vegetables. This is the only observation I have made in recent years. Here [in this household] we are now producing lettuces and cabbage and its going well. It was not in our habits but … it’s important because it improves the (household) nutrition diet…” (21NNA1_villagechief, 66)*
Restricting meal numbers/frequency	Households have moved from three meals a day to two meals a day. Furthermore, there is a limitation of any other food consumption throughout the day and in-between meals. Very young children are often exempt from this restriction, still receiving small amounts of food for breakfast or daytime snacks.	*“Yes, when the production is favorable, we can obviously feed our families well. We can eat morning, noon and evening… These days, generally, it is mainly noon and evenings (that we eat)” (4BRS_regionalChief, 66)*
Reducing meal diversity	Households have reduced the diversity and variety of the meals consumed by strictly eating the gains from their own production and no longer purchase foods to bring variety to meals and to reduce or eliminate “luxury” food items.	*“…before we were financially better off, we made fish soup often but today we do it rarely… before we varied food every 2 days, but that was a long time ago. Today, every day, it’s Tô. Today, the children know that financially it’s not going well, [since meals] are only Tô” (28TSI_farmer, 49)*
Reducing meal size/portion	Households have systematically reduced the quantity of food prepared per meal, primarily by using fewer tin/cans of grains/cereals, then habitually, to make a meal.	*“In one day, you prepare two boxes of millet, midday and in the evening as a meal. Now the [agricultural] season has been bad. You were used to a meal of two boxes a day and you are told to consume one box a day, you know that this is a real difficulty. You know you will no longer be full“ (30TNI2_farmer, 44)*
Purchasing government subsidized food	Households who have the possibility to purchase government subsidized food have reported doing so, but pointed out that such food is neither often available nor is the information of their availability systematically distributed to every household.	*“Yes, I have already purchased corn at SONAGES [The National Society for Food Security Stock Management]. They have come and deposit stock in Siélla, it was for our area. I went to Siélla to get some…” (8DKM_farmer, 47)*
Preemptively purchasing grains/cereals	Household heads and their spouses purchased cereals and grains at the first sign of an insufficient harvest, during the harvest season. The purchased grains were then stored to be utilized in case of shortages. For these households, preemptive purchasing, the early purchase of grains/cereals allowed them to effectively avoided the high prices late in the year, when many households have consumed all their harvest and must turn to market sold grains/cereals to sustain for the remainder of the year. At this time prices are high because demand is also high but available supply of grains/cereals is low.	*“When I notice [during harvest] that there will be a lack of food, I purchase some and store it. When the little that we have cultivated is consumed, we consume what I bought and stored. I purchase the food early, before it gets more expensive” (11DNA_farmer, 55)*
